# Plasma Neurofilament Light Chain Is Associated with Cognitive Functions but Not Patient-Reported Outcomes in Multiple Sclerosis

**DOI:** 10.3390/neurolint17090144

**Published:** 2025-09-09

**Authors:** Valerio Nicolella, Federica Novarella, Fabrizia Falco, Carmela Polito, Rosa Sirica, Evelina La Civita, Vincenzo Criscuolo, Giuseppe Corsini, Antonio Luca Spiezia, Alessia Castiello, Antonio Carotenuto, Maria Petracca, Roberta Lanzillo, Giuseppe Castaldo, Vincenzo Brescia Morra, Daniela Terracciano, Marcello Moccia

**Affiliations:** 1 Department of Neurosciences, Reproductive and Odontostomatological Sciences, Federico II University of Naples, 80131 Naples, Italy; 2 Multiple Sclerosis Unit, Policlinico Federico II University Hospital, 80131 Naples, Italy; 3 Department of Translational Medical Sciences, Federico II University of Naples, 80131 Naples, Italy; 4 Department of Molecular Medicine and Medical Biotechnology, Federico II University of Naples, 80131 Naples, Italy; vincenzocris46@gmail.com (V.C.); giuseppe.castaldo@unina.it (G.C.); 5 Department of Human Neuroscience, Sapienza University of Rome, 00185 Rome, Italy; 6 Centre for Advanced Biotechnology (CEINGE), 80131 Naples, Italy

**Keywords:** multiple sclerosis, neurofilament, cognitive, patient-reported outcomes

## Abstract

Objective: We aimed to explore associations between plasma neurofilament light chain (pNfL) and cognition through patient-reported outcomes (PROs) in multiple sclerosis (MS). Methods: In this cross-sectional study, we included 211 people with MS (PwMS) and collected data from pNfL (fully automated chemiluminescent enzyme immunoassay), EDSS, education, cognition (the Symbol Digit Modalities Test (SDMT), California Verbal Learning Test-II (CVLT II), and Brief Visuospatial Memory Test–Revised (BVMT-R)), the Modified Fatigue Impact Scale (MFIS), Beck Depression Inventory (BDI-II), Beck Anxiety Inventory (BAI), and Pittsburgh Sleep Quality Index (PSQI). Results: On multivariate linear regression models, higher educational attainment was significantly associated with lower pNfL (high school: Coeff = −0.22, 95% CI = −0.41 to −0.04, *p* = 0.019; university: Coeff = −0.22, 95% CI = −0.42 to −0.02, *p* = 0.030). In logistic regression models, the likelihood of having pNfL levels above normal thresholds increased by 56% for each one-point increment in the EDSS score (OR = 1.56, 95% CI = 1.23 to 1.98, *p* < 0.001) and was 2.5 times greater in individuals with impaired SDMT (OR = 2.50, 95% CI = 2.20 to 5.21, *p* = 0.014). No statistically significant associations were observed between pNfL and CVLT-II, BVMT-R, BDI-II, MFIS, BAI, or PSQI. Conclusions: Neuro-axonal damage in people with MS manifests clinically as increased disability and reduced attention and processing speed. However, these effects may be mitigated by greater brain resilience, as suggested by the protective role of higher educational attainment. The PROs assessed in this study showed no significant associations with pNfL levels, possibly due to measurement errors and heterogeneity, with limited sensitivity to neuro-axonal damage.

## 1. Introduction

Multiple sclerosis (MS) is characterized by a combination of clinical features, including cognitive impairment, fatigue, sleep issues, anxiety, and depression. These symptoms are often referred to as “invisible” and affect individuals’ ability to function in social and working life [1,2]. Cognitive impairment affects up to 70% of people with MS (PwMS) at the time of clinical onset and is thought to be caused by a progressive accumulation of functional disconnection and of neuro-axonal damage, with cognitive reserve capacity failing to counteract brain damage [1]. These deficits span multiple domains, most notably processing speed, but also episodic memory and executive, verbal, and visuospatial functions [2]. Other invisible symptoms of MS, such as fatigue, depression, and anxiety, are directly reported by PwMS, sometimes using specific questionnaires and scales, and are then referred to as patient-reported outcomes (PROs). PROs have been gaining importance in clinical trials and observational studies, because they directly reflect an individual’s perceptions of disease impairments [3,4,5]. However, the pathological background of PROs is largely unknown, though neuro-axonal damage could provide a significant contribution. Sleep impairments have more recently emerged as a clinically significant issue in MS, affecting individuals’ activities in daily living, and, in turn, might be associated with increased neuro-axonal damage based on evidence in the general population [6,7]. Fatigue in MS is likely multifactorial but holds structural correlates, suggesting that neuro-axonal loss could be involved, especially in more advanced stages [8,9,10,11].

Nowadays, blood-based biomarkers are non-invasive tools allowing us to study disease pathology and related clinical expressions over the course of follow-up. In particular, neurofilament light chain (NfL) is a neuronal cytoskeletal protein that is released into CSF and blood in the case of neuro-axonal injury [12]. In MS, NfL levels increase with relapses, active MRI lesions, and disability progression, and decrease following effective treatment [13,14]. A limited number of studies showed associations between higher NfL levels and worse cognitive performance in MS [15,16,17,18,19], thus confirming their association with neuro-axonal damage. However, associations between NfL and PROs have been explored by very few studies and are not guaranteed due to contradictory results and the inclusion of a limited number of PROs in highly selected populations [20,21,22,23].

As such, we aim to confirm the associations between plasma NfL (pNfL) and cognitive performance in MS, and to explore potential associations with a wide range of PROs using age-adjusted NfL cut-offs [24].

## 2. Methods

### 2.1. Study Design and Population

This retrospective study was based on clinical and laboratory data routinely collected at the MS Clinical Unit of the Federico II University Hospital in Naples, Italy. Ethical approval was obtained from the Federico II Ethics Committee (protocol no. 332/21, 16 December 2021). All participants provided informed consent for the use of anonymized data in compliance with the European General Data Protection Regulation (GDPR, EU 2016/679). The study was conducted in accordance with Good Clinical Practice guidelines and the Declaration of Helsinki. Inclusion criteria were as follows: (1) MS diagnosis according to the 2017 McDonald criteria, regardless of relapse or progressive aspects, and (2) availability of demographic and clinical variables, cognitive assessment, PROs, and pNfL within the last 3 months.

Exclusion criteria were as follows: (1) a history of significant medical illnesses (including kidney dysfunction), fever, or substance abuse in the 30 days before study entry; (2) other major systemic, psychiatric or neurological diseases; (3) relapses, evidence of MRI activity, or corticosteroid treatment in the 30 days before and after sample collection; and (4) concomitant medications possibly affecting cognitive or psychological function (i.e., antidepressants, antipsychotics, and benzodiazepine).

### 2.2. Demographics and Clinical Variables

For each participant, demographic and clinical data were collected, including age, sex, and educational level (based on highest level of attainment). Body mass index (BMI) was calculated from measured height and weight. Additional variables included smoking status (categorized as smoker or never smoker), presence of cardiovascular comorbidities (such as hypertension, hypercholesterolemia, diabetes, atrial fibrillation, stroke, coronary artery disease, and related medications), disease duration (from reported onset to assessment), an Expanded Disability Status Scale (EDSS) score, a descriptor of disease progression (relapsing or progressive), and any current disease-modifying therapy (DMT).

### 2.3. NfL Measurement

Fasting blood samples were centrifuged within 3 h after they were drawn at 1100 rpm × 10 min, aliquoted into polypropylene tubes, and stored at −80 °C. pNfL levels were evaluated using a fully automated chemiluminescent enzyme immunoassay (LUMIPULSE^®^, Fujirebio, Tokyo, Japan), expressed as picogram per milliliter (pg/mL). We preferred the use of plasma over serum due to the faster processing time, higher yield, and reduced risk of interference from clotting (especially when multiple biomarkers are performed at the same time) [25,26]; we previously demonstrated that the levels of pNfL and sNfL measured using this methodology provide similar and highly related results [26].

Based on pNfL values and age group (5 to <18, 18 to <50, 50 to <60, 60 to <70, and >70 years), we then classified PwMS as below or above normal values using previously established cutoffs [24,25,26]. The raw values of pNfL were also transformed into a logarithmic function (log-pNfL) for subsequent analysis to reduce the skewness of the distribution [19].

### 2.4. Cognitive Variables

Cognitive performance was evaluated using the Italian adaptation of the Brief International Cognitive Assessment for Multiple Sclerosis (BICAMS) battery [27]. This tool encompasses tests targeting attention, information processing speed, and working memory—assessed via the Symbol Digit Modalities Test (SDMT)—as well as verbal and visuospatial memory, evaluated through the California Verbal Learning Test-II (CVLT-II) and the Brief Visuospatial Memory Test–Revised (BVMT-R), respectively [27]. All scores were adjusted for age, sex, and educational level using established Italian normative data [28], with scores below 35 considered indicative of cognitive impairment.

### 2.5. PROs

On the same day as cognitive assessment, each patient filled in the Modified Fatigue Impact Scale (MFIS) (with scores ≥ 38 indicating fatigued individuals, and additional subscores included for cognitive, physical, and psychosocial fatigue) [29]; the Beck Depression Inventory (BDI-II) (with scores ≥ 14 indicating mild-to-severe depression) [30]; the Beck Anxiety Inventory (BAI) (with scores ≥ 8 indicating mild-to-severe anxiety); and the Pittsburgh Sleep Quality Index (PSQI) (with scores > 5 indicating sleep disorders) [31,32].

### 2.6. Statistical Analyses

Descriptive statistics were calculated for all relevant variables. Continuous data—such as age, body mass index (BMI), disease duration, and scores from the SDMT, CVLT-II, BVMT-R, MFIS, PSQI, BDI-II, and BAI—are presented as means with standard deviations. The EDSS is reported as the median with a range. Categorical variables—including age group, sex, educational attainment, smoking status, presence of cardiovascular comorbidities, disease progression descriptor, and type of DMT—are summarized as counts and percentages.

For each demographic (age, sex, education, smoking, and presence of cardiovascular comorbidities), clinical (disease duration, EDSS, and descriptor of disease progression), cognitive (SDMT impairment, CVLT impairment, and BVMT impairment), and PRO variable (MFIS impairment, BDI impairment, BAI impairment, and PSQI impairment), we ran multivariate linear regression models to evaluate associations with log-transformed pNfL values (log-pNfL, as dependent variable), and then used multivariate logistic regression models to evaluate associations with pNfL above or below (reference in the statistical model) normality values (as the dependent variable). This dual approach was preferred to test the validity of suggested (but not widely accepted or applied) cut-offs of pNfL, while not risking missing significant linear associations.

Covariates for all models were age, sex, education, smoking, presence of cardiovascular comorbidities, EDSS, and, in separate models, BMI as well (available for a subset of the population). Statistical results are presented as coefficients (Coeff), odds ratios (ORs), 95% confidence intervals (95% CIs), and *p*-values, as appropriate. The distribution of variables and model residuals was assessed using both graphical and statistical methods. Analyses were conducted using Stata version 15.0 (StataCorp, College Station, TX, USA). Statistical significance was defined as a *p*-value less than 0.05.

#### Power Calculation

Our sample of 211 patients is sufficient to reach statistical significance at Coeff = 0.05 using regression models, with 5% alpha and 90% power.

## 3. Results

### 3.1. Study Population

We included 211 PwMS (age 44.7 ± 12.2 years; 62.56% females; and pNfL 12.32 ± 11.35 pg/mL). Demographic, clinical, cognitive, PROs, and laboratory variables are presented in Table 1.

### 3.2. Demographic and Clinical Correlates

**Figure 1:**
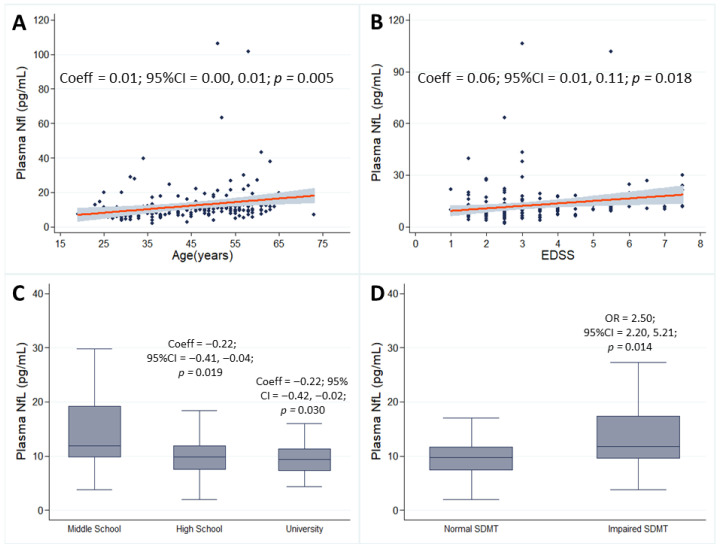
Scatter plots show associations between plasma neurofilament light chain (pNfL) and age (**A**) and EDSS (**B**) (gray shades represent confidence intervals). Box and whisker plots show associations between pNfL and education (**C**) and SDMT (**D**). Coefficients (Coeff), odds ratios (ORs), 95% confidence intervals (95% CIs), and *p* values are presented for significant associations.

On multivariate linear regression models, higher pNfL levels were associated with older age (Coeff = 0.01; 95% CI = 0.00, 0.01; *p =* 0.005) (Figure 1A), and higher EDSS (Coeff = 0.06; 95% CI = 0.01, 0.11; *p =* 0.018) (Figure 1B); in addition, lower pNfL levels were associated with higher educational attainments (high school: Coeff = −0.22; 95% CI = −0.41, −0.04; *p =* 0.019; university: Coeff = −0.22; 95% CIs = −0.42 and −0.02; *p =* 0.030) (Figure 1C); no associations were found for sex, smoking, presence of cardiovascular comorbidities, disease duration, descriptor of disease progression, or DMT. On multivariate logistic regression models, each year of age was associated with a 3% lower probability of pNfL above normal values (OR = 0.97; 95% CI = 0.94, 0.99; *p =* 0.04), and each EDSS point was associated with a 56% higher probability of pNfL above normality values (OR = 1.56; 95% CI = 1.23, 1.98; *p* < 0.001); no associations were found for sex, education, smoking, presence of cardiovascular comorbidities, disease duration, descriptor of disease progression, or DMT (Table 2).

When including BMI among covariates, we confirmed associations between higher pNfL and older age (OR = 0.95; 95% CI = 0.91, 0.99; *p =* 0.17) and higher EDSS (Coeff = 0.08; 95% CI = 0.02, 0.14; *p =* 0.012; OR = 1.75; 95% CI = 1.23, 2.49; *p* = 0.002); we also confirmed associations between lower pNfL and higher educational attainments (high school: Coeff = −0.24; 95% CI = −0.45, −0.03; *p =* 0.027; university: Coeff = −0.24; 95% CI = −0.47, −0.01; *p =* 0.042).

Table 2 shows coefficients (Coeff), odds ratios (ORs), 95% confidence intervals (95% CIs), and *p* values from mixed-effect regression models, including log-pNfL values as dependent variables, and different demographic (age, sex, education, smoking, and presence of cardiovascular comorbidities) and clinical variables (disease duration, EDSS, and descriptor of disease progression), in turn, as independent variables; covariates were age, sex, education, smoking, presence of cardiovascular comorbidities, and EDSS. Significant results (*p* < 0.05) are reported in bold.

### 3.3. Cognitive and PRO Correlates

On multivariate linear regression models, no associations were found for SDMT, CVLT, BVMT, MFIS, and its subscores BDI, BAI, PSQI, and pNfL. In multivariate logistic regression analyses, people with multiple sclerosis (PwMS) exhibiting impaired performance on the SDMT had a 2.5-fold higher likelihood of presenting pNfL levels above the normal range (OR = 2.50; 95% CI: 2.20–5.21; *p* = 0.014). (Figure 1D). No associations were found for CVLT, BVMT, MFIS, and its subscores BDI, BAI, and PSQI (Table 3).

When including BMI among covariates, we confirmed associations between higher pNfL and lower SDMT (Coeff = 0.28; 95% CI = 0.11, 0.45; *p =* 0.001; OR = 5.63; 95% CI = 2.02, 15.72; *p =* 0.001).

The table shows coefficients (Coeff), odds ratios (ORs), 95% confidence intervals (95% CIs), and *p* values from mixed-effect regression models, including log-pNfL values as dependent variables and different cognitive variables (SDMT impairment, CVLT impairment, and BVMT impairment) and different PROMs (MFIS impairment, BDI impairment, BAI impairment, and PSQI impairment), in turn, as independent variables; covariates were age, sex, education, smoking, presence of cardiovascular comorbidities, and EDSS. Significant results (*p* < 0.05) are reported in bold.

## 4. Discussion

We showed that PwMS with impairments in attention and processing speed (i.e., SDMT) have a higher pNfL. On the contrary, higher educational attainments (i.e., above high school graduation) are associated with a lower pNfL. Also, we confirmed the associations between higher pNfL and older age and worse disability (i.e., EDSS). However, we failed to find significant associations (and numerical trends) between pNfL and PROs.

Looking at cognitive features, PwMS with lower educational attainment and impaired SDMT had higher pNfL. The association between higher pNfL and lower performance on attention and processing speed tasks has already been established by a number of studies, mostly conducted with people with progressive phases of MS [15,16,17,18,19]. For instance, in progressive MS, Gaetani and colleagues found that serum NfL (sNfL) was predictive of subsequent cognitive decline, and Williams and colleagues found similar associations in a sub-analysis of a randomized controlled trial [17,18]. On the contrary, our population consisted of PwMS with a predominantly relapsing course, thus expanding this association within the spectrum of MS. Cognitive impairment, in particular, lower SDMT scores have been previously associated with the risk of disease progression, as is the case for higher pNfL levels [27,33,34]. As such, the association between pNfL and SDMT supports existing evidence that cognitive impairment in MS is a clinical expression of neuro-axonal injury. On the other hand, there have been conflicting results on the associations between other cognitive functions and pNfL in MS [25]. Cognitive changes may evolve with advancing age and disease duration, increasingly affecting domains commonly impacted by aging, such as memory (assessed by CVLT and BMVT), rather than those typically linked to MS, like processing speed [25,35].

The association between pNfL and educational attainment is novel, though not completely unexpected. Based on our results, high school and university graduates with MS have a lower probability of pNfL above normality values, compared with PwMS with lower educational attainments. Education reflects early intellectual enrichment and could be considered a raw index of cognitive reserve [36]. Education mediates lifetime brain volume (i.e., brain reserve), which, along with cognitive reserve, affects the probability of cognitive progression in MS [37,38]. In keeping with this, a recent study showed that a larger brain reserve is associated with delayed clinical onset of MS, including much more elevated compensatory abilities [39]. Overall, our results suggest that early intellectual enrichment can affect long-term trajectories of neuro-axonal injury, thus opening new perspectives on cognitive training in MS. However, we cannot exclude the fact that lower educational attainment carries biases from other social determinants of health [40].

We did not find any significant association or numerical trend between pNfL levels and PROs reflecting fatigue, depression, anxiety, and sleep disorders. Considering that our sample has sufficient power to detect meaningful effects in regression analyses, this negative result could be attributed to multiple factors, including the lack of biological associations between NfL and explored PROs [13,14]. In keeping with this, some previous studies failed to find significant associations [20,21]. For instance, Aktas and colleagues failed to find an association between sNfL levels and fatigue, anxiety, and depression, but included a relatively small sample (45 clinically stable PwMS) [20]. In another study including 38 patients diagnosed with clinically isolated syndrome and relapse-remitting MS (RRMS), Håkansson et al. reported that fatigue was not correlated with NfL levels. Instead, fatigue was significantly associated with anxiety and health-related quality of life, suggesting a complex interplay among various PROs independent of neuro-axonal injury [21]. In a longitudinal study on relapsing and progressive PwMS, Galetta and colleagues found that baseline sNfL correlated with baseline MS quality of life (MSQoL) physical composites, while both baseline and follow-up sNfL correlated with MSQoL physical and social functioning limitations, which, in turn, were associated with brain atrophy [23]. Taken together, it is possible that the PROs explored (fatigue, depression, anxiety, and sleep disorders) reflect a combination of different mechanisms and do not necessarily imply neuro-axonal damage but rather depend on individuals’ perceptions of the disease and its symptoms. [23,41]. In keeping with this, it is possible that the PROs we have used, while well validated in MS populations and widely utilized in both clinical trials and observational studies, are not sufficiently sensitive to MS pathophysiology and related neuro-axonal injury [10,42,43], and thus have psychometric limitations. For instance, the MFIS has shown psychometric weaknesses in its subscales, potentially undermining meaningful interpretation; the BDI-II and BAI emphasize somatic symptoms, which can inflate scores in participants with physical illness; and the PSQI’s single-factor structure and reliance on self-report may fail to capture sleep’s complexity and correlate only modestly with objective measures. [44,45,46]. In this scenario, measurement error and heterogeneity related to explored PROs could be responsible for the lack of significant associations. The development of new PROs should be strongly encouraged, and their validation towards pathologically meaningful biomarkers should be considered [3,47].

Looking at demographics and clinical features, we confirmed a significant association between higher pNfL and worse disability (EDSS). A number of previous studies have highlighted this association [12,48], and, in keeping with this, we have consistently included the EDSS among our covariates. Also, we found higher pNfL in relation to older age, which, again, is a well-established association [49]. Intriguingly, when deriving age-adjusted cut-offs, older PwMS showed a lower probability of having pNfL above normality values, possibly reflecting neuro-axonal loss in earlier disease stages with subsequent ceiling effects [49]. BMI and, in general, hemodilution can affect NfL levels, and, in our study, we also included BMI in the subgroup with available data in the absence of significant statistical changes [50].

Study limitations include the cross-sectional design, which prevents the assessment of temporal changes. Longitudinal studies are warranted to evaluate how variations in pNfL correlate with changes in cognitive function and patient-reported outcomes (PROs) [20]. Of note, pNfL was independently associated with EDSS and SDMT, but we could not exclude the fact that cognitive reserve affected both outcomes, while only educational attainment was available [51]. In particular, our cross-sectional design cannot rule out the possibility that the association between pNfL and cognitive reserve is merely coincidental, rather than causal [20]. Education may not fully capture the cognitive reserve construct, and additional factors, including occupation, socioeconomic status, and involvement in mentally stimulating activities, should be considered in the future to further deepen the association between axonal damage and cognitive reserve [52]. Future research should also consider broader outcome measures, including comprehensive cognitive assessments, relevant biomarkers, and advanced MRI evaluations, to further elucidate the relationship between pNfL levels and the heterogeneity of MS clinical manifestations [47,53].

In conclusion, our results confirmed that more neuro-axonal damage can express clinically worse disability and worse attention and processing speed in MS. Also, we showed that higher educational attainment is associated with lower pNfL, as well as increased resilience to neuro-axonal damage. We failed to demonstrate associations between pNfL and PROs, thus suggesting that we need to develop, validate, and apply novel measures that are not only meaningful for PwMS and their daily functioning but that also reflect MS pathophysiology.

## Figures and Tables

**Table 1:** Demographic, clinical, cognitive, PRO, and laboratory variables.

	*n* = 211
Age, years	44.7 ± 12.2
Age group 18–50, *n* (%) 50–60, *n* (%) 60–70, *n* (%) 70+, *n* (%)	132 (62.56%) 58 (27.49%) 20 (9.48%) 1 (0.47%)
Sex, females (%)	138 (65.4%)
Education, years Middle School (%) High School (%) University (%)	15.53 ± 9.10 47 (22.27%) 102 (48.34%) 62 (29.38%)
Cardiovascular comorbidity (%)	47 (22.27%)
Ever Smoking (%)	30 (14.22%)
BMI (*n* = 139)	24.60 ± 4.7
	
Disease duration, years	13.13 ± 3.58
	
EDSS, median (range)	2.5 (1.0–7.5)
	
*Descriptor of disease progression* Relapsing Progressive	170 (80.53%) 41 (19.47%)
	
*Current DMT duration(years)* *DMT group* No DMT Oral DMTs Monoclonal antibody DMTs Injective DMTs	4.01 *±* 4.20 5 (2.37%) 137 (64.97%) 53 (25.12%) 16 (7.58%)
	
SDMT Impaired SDMT (%)	43.53 ± 12.24 55 (26.07%)
CVLT Impaired CVLT (%)	42.76 ± 13.42 65 (30.81%)
BVMT Impaired BVMT (%)	42.30 ± 11.39 60 (28.44%)
MFIS cognitive MFIS physical MFIS psychosocial MFIS total Impaired MFIS (%)	8.78 ± 9.76 10.09 ± 10.39 1.65 ± 3.31 20.53 ± 21.08 46 (21.80%)
	
BDI Impaired BDI (%)	7.79 ± 10.23 49 (23.22%)
BAI Impaired BAI (%)	6.97 ± 13.31 38 (24.84%)
PSQI Impaired PSQI (%)	3.32 ± 4.33 27 (17.65%)
	
*NfL* pNfL (pg/mL) pNfL above normality (%)	12.32 ± 11.35 71 (33.75%)

**Table 2:** NfL and demographic and clinical correlations.

	NfL Cut-Offs from Simrén and Colleagues [24]					
				95% CI		
	Normal *n* = 140	Higher than Normal *n* = 71		Lower	Upper	*p* Value
Age	45.34 ± 13.19	43.31 ± 9.78	Coeff 0.01 OR 0.97	0.00 0.94	0.01 0.99	**0.005** **0.04**
Sex females vs. males	Males 44 (60.27%)	Males 29 (39.73%)	Coeff 0.00 OR 1.25	−0.14 0.66	0.14 2.36	0.95 0.49
Education class middle school (reference) high school University	27 (19.29%) 70 (50.00%) 43 (30.71%)	20 (28.17%) 32 (45.07%) 19 (26.76%)	reference Coeff−0.22 OR 0.64 Coeff−0.22 OR 0.78	−0.41 0.27 −0.42 0.31	−0.04 1.53 −0.02 2.00	**0.019** 0.319 **0.030** 0.611
Cardiovascular comorbidity	34 (24.29%)	13 (18.31%)	Coeff 0.10 OR 0.84	−0.08 0.37	0.27 1.90	0.270 0.686
Smoking	15 (10.71%)	15 (21.13%)	Coeff 0.03 OR 2.19	−0.15 0.95	0.22 5.03	0.710 0.065
EDSS	2.5 (1–7.0)	3 (1–7.5)	Coeff 0.06 OR 1.56	0.01 1.23	0.11 1.98	**0.018** <**0.001**
Disease duration	15.31 ± 9.48	15.96 ± 8.35	Coeff 0.00 OR 1.02	−0.01 0.98	0.01 1.07	0.590 0.382
Relapsing vs. progressive	Progressive 24 (17.27%)	Progressive 17 (23.94%)	Coeff 3.19 OR 0.56	−8.02 0.20	1.64 1.53	0.194 0.259

**Table 3:** NfL and cognitive and PRO correlates.

	NfL Cut-Offs from Simrén and Colleagues [24]					
				95% CI		
	Normal *n* = 140	Impaired *n* = 71		Lower	Upper	*p* Value
SDMT Impaired vs. Normal	Impaired 28 (20.00%)	Impaired 27 (38.03%)	Coeff 0.29 OR 2.50	0.11 1.20	0.45 5.21	0.289 **0.014**
CVLT Impaired vs. Normal	Impaired 42 (30.00%)	Impaired 23 (32.39%)	Coeff −0.06 OR 0.87	−0.21 0.42	0.1 1.77	0.476 0.697
BVMT Impaired vs. Normal	Impaired 38 (27.14%)	Impaired 22 (30.00%)	Coeff −0.33 OR 0.94	−0.19 0.45	0.13 1.94	0.682 0.858
MFIS Cognitive Fatigue	8.70 ± 10.11	8.93 ± 9.09	Coeff −0.00 OR 1.00	−0.01 0.96	0.00 1.03	0.253 0.797
MFIS Physical fatigue	10.44 ± 11.01	9.41 ± 8.92	Coeff −0.00 OR 0.98	−0.12 0.95	0.00 1.00	0.119 0.171
MFIS Psychological Fatigue	1.73 ± 2.40	1.51 ± 2.12	Coeff −0.03 OR 0.90	−0.06 0.78	0.00 1.05	0.064 0.180
MFIS Total Fatigue Impaired vs. Normal	Impaired 32 (22.86%)	Impaired 14 (19.72%)	Coeff −0.50 OR 0.07	−0.22 0.30	0.12 1.50	0.556 0.333
BDI-II Impaired vs. Normal	Impaired 33 (23.57%)	Impaired 16 (22.54%)	Coeff −0.02 OR 0.99	−0.19 0.46	0.14 2.11	0.784 0.974
BAI Impaired vs. Normal	Impaired 25 (24.51%)	Impaired 13 (25.49%)	Coeff −0.05 OR 1.12	−0.23 0.47	0.13 2.68	0.563 0.802
PSQI Impaired vs. Normal	Impaired 18 (17.65%)	Impaired 9 (17.65%)	Coeff −0.18 OR 1.03	−0.40 0.30	0.41 3.51	0.109 0.961

## Data Availability

The original contributions presented in this study are included in the article. Further inquiries can be directed to the corresponding author.
